# EVALUATING FRAILTY INDEX INTEGRITY: INSIGHTS FROM AN INTERNATIONAL NETWORK STUDY

**DOI:** 10.1093/geroni/igae098.3966

**Published:** 2024-12-31

**Authors:** Robert Cavanaugh, Chen Yanover, Chelsea Wong, Tal El-Hay, Ariela Orkaby, Maytal Bivas-Benita, Pinchas Akiva, Brianne Olivieri-Mui

**Affiliations:** Northeastern University, South Portland, Maine, United States; KI Institute, Tel Aviv, Israel; Beth Israel Deaconess Medical Center, Brookline, Massachusetts, United States; KI Institute, Tel Aviv, Israel; Brigham and Women’s Hospital, Boston, Massachusetts, United States; KI Institute, Tel Aviv, Israel; KI Institute, Tel Aviv, Israel; Northeastern University, Boston, Massachusetts, United States

## Abstract

Deficit-accumulation Frailty indices (FIs) are a key tool in population-level translational geroscience approaches to understanding function, health, and aging. International applications of Fis have been enabled by the uptake of common data models (CDMs) but the validity of such applications is not guaranteed. We compared frailty prevalence using two validated electronic health record based FIs - the United States (US)-based Veterans Affairs FI (VAFI) and United Kingdom (UK)-based electronic FI (eFI) across 5 Observational Medical Outcomes Partnership CDM databases. Frailty was calculated by age for patient-records 40+ years old with 1+ years of data in the 1) US All of Us database (n=211,568), 2) US IQVIA Pharmetrics+ (n=5,292,854), 3) UK IMRD-THIN (n=3,080,557), 4) UK IMRD-EMIS (n=843,928), 5) UK BioBank (n=209,566). Code and results are available in an open-source R package and interactive website. The VAFI identified higher prevalence of frailty in US databases (All of US: VAFI 10.3% v eFI 2.5%, Pharmetrics+: 9.6% v 2%) while the eFI identified higher prevalence in UK databases (IMRD-THIN: < 0.003% v 0.08%, IMRD-EMIS: 0.09% v 0.4%, UK BioBank: 0.03% v 0.1%). eFI prevalence was lower than previously reported (2.2% v 15-20%, >65 years old; Clegg et al., 2016). FIs appear to be dependent on their development context, limiting external validity for international network studies. FIs must be vetted for consistency and validity when used across CDM sources if they are to contribute to the study of health and aging at scale. Future work will extend this comparison to additional international data sources.

